# Thinking Together, Working Apart: Leveraging a Community of Practice to Facilitate Productive and Meaningful Remote Collaboration

**DOI:** 10.34172/ijhpm.2020.122

**Published:** 2020-07-12

**Authors:** Mark Embrett, Rebecca H. Liu, Katie Aubrecht, Andriy Koval, Jonathan Lai

**Affiliations:** ^1^Health Program, St. Francis Xavier University, Antigonish, NS, Canada.; ^2^Women’s College Hospital Institute for Health System Solutions and Virtual Care, University of Toronto, Toronto, ON, Canada.; ^3^Department of Sociology, St. Francis Xavier University, Antigonish, NS, Canada.; ^4^University of Central Florida, Orlando, FL, USA.; ^5^Canadian Autism Spectrum Disorder Alliance (CASDA), Toronto, ON, Canada.

**Keywords:** Remote Collaboration, Social Distancing, Community of Practice, Communication Technology, Training Modernization

## Abstract

Considering the coronavirus disease 2019 (COVID-19) pandemic, scholars were encouraged to cease collocated meetings. Many researchers have turned to remote collaboration to continue group-based projects. This paper focuses on the structure, processes, and outcomes that a group of physically distanced, embedded researchers used to collaborate across Canada to produce research outputs prior to the pandemic. The intent of this paper is to provide an overview of mechanisms that can facilitate meaningful and productive remote collaboration using online and digital technologies as a feasible and effective alternative mode of communication for research teams.

## Introduction

In Spring 2020 global efforts to ‘flatten the curve’^1^ of the coronavirus disease 2019 (COVID-19) pandemic included the postponement or cancellation of many in-person gatherings around the world.^2^ For instance, in Canada, guidance from the government included the practice of social distancing, which involves ‘ *minimizing close contact with others during the peak of an outbreak.’*^3^ Similarly, the World Health Organization (WHO) provided fundamental mitigation and suppression strategies to reduce COVID-19 mortality and healthcare demand, which included social distancing and working from home in isolation.^4^ In response, researchers began reducing, if not eliminating, collocated research activities. However, even before COVID-19, researchers from the natural, social and health sciences were gravitating towards interdisciplinary, trans-institutional, and cross-geographic collaboration.^5^ Taken all together, this suggests a growing interest and need to improve researchers’ capabilities to collaborate. Remote collaboration using online and digital technologies is one approach to facilitate interdisciplinary research, while still maintaining meaningful and productive working relationships.

This paper describes the authors’ collective experiences in remote collaboration as a cohort of the Canadian Institutes of Health Research (CIHR) Health System Impact Fellowship (HSIF).^6^ Specifically, the experiences focus on working together as a community of practice (CoP) during the inception, design, execution, analysis, and write-up of an electronic Delphi (eDelphi) study, identifying the key success criteria of the embedded research fellowship.^7^ The purpose of describing these experiences is to provide a framework to work within, as well as a set of resources and recommendations for the use of technology for researchers interested in remote collaboration. A CoP framework was adopted as an effective guide to outlining the formation and maintenance of remote collaboration via three distinct facets of support^8^:

1. *Structure:* Defines the scope of a CoP by scaffolding its activities around its identity and vision. This also helps to establish the boundaries needed to maintain momentum towards goals.
2. *Process:* Establishes the coordination, transparency, and negotiability of the CoP and its activities, milestones, and intentions, including how to use technology, how to collaborate, and achieve objectives.
3. *Outcome:* Provides recommendations for future cohorts, as well as those interested in establishing and promoting a CoP using remote collaboration. Aids the dissemination of eDelphi study at HSIF National Cohort Retreat and promotes the knowledge and adaptation of remote collaboration processes (current article).

Considering the COVID-19 pandemic, this overview of remote collaboration mechanisms may serve to be timely in facilitating, while also producing, interdisciplinary collaborative work. These resources can also be extended for future circumstances, post-COVID-19, as an alternative means of communication across distances, and when resources and capacity are limited.

## Background on the CIHR Health System Impact Fellowship

In 2015, CIHR launched the HSIF – an experiential training program^9^ aimed at harnessing the talent of PhD-level researchers for the improvement of health system performance.^6^ The fellowship aimed to create a community of leaders committed to evidence-based improvement of Canadian healthcare. A special issue in Healthcare Policy^10^ reports on the early outcomes of the program based on the inaugural cohort. Below, we describe how members of this cohort functioned as a CoP to carry out the eDelphi project through remote collaboration.

###  1. Structure: A Community of Practice

The CoP structure is a tool to support education and promote learning as a social endeavor.^11^ A CoP provides an enduring structure for groups to coalesce around shared interests and ideas, using activities characterized by *mutual engagement*, *joint enterprise*, and a *shared repertoire.*^12^ ‘Mutual engagement’ describes the interaction among members that strengthens their connections and leads to shared understanding. ‘Joint enterprise’ refers to the process of engagement where members work towards a common goal. Finally, a ‘shared repertoire’ captures the use of communal resources that facilitate group engagement.

One approach to overcoming operational challenges of a CoP involves maintaining an informal, flexible mode of collaboration using a “team-like” organization that fosters group identity and interest, which then improves spontaneous communication and idea generation.^13^ Accommodating these needs led the CoP to develop a self-governed process, along with various forms of collaboration (detailed below).

###  2. Process: The Use of Digital Services to Facilitate Collaboration

Throughout its course, the project included several forms of mutual engagement, supported by various forms of digital services (Table). This section describes: (*a*) the management, (*b*) communications, and (c) the output processes along with the various corresponding activities of the CoP of Fellows.

**Table:** Description of How Digital Services Were Used by the CoP of Fellows

**Shared Activities for Fellowship Collaboration**	**How Technology Was Used to Maintain and Enhance Collaboration**		
	**CoP Management; Coordinating and Scheduling Meetings and Tasks (Doodle, WhenIsGood ?)**	**Communications and social Interactions; Use of Synchronous and Asynchronous Technologies (Zoom, Skype, BlueJeans )**	**Output platform; Online editing and storage software (Google Documents, Google Drive )**
Sustained mutual relationships and support (eg, common experiences and interests, like peer mentorship)		X	X
Shared ways of engaging together (eg, sharing ideas, experiences)		X	X
Information sharing and promotion of ideas and interests (eg, conferences, grant applications, etc)		X	X
Interactions on an ongoing process (eg, time management, flexible, pragmatic and efficient, expectation management)	X	X	
Knowing what others know, what they can do, and how they can contribute (eg, how we share our ideas and collaborate)		X	X

Abbreviation: CoP, community of practice.

####  (a) CoP Management; Coordinating and Scheduling Meetings and Tasks 

####  Facilitation Strategies

Several online scheduling services, such as Doodle (https://doodle.com/) or WhenIsGood (https://whenisgood.net/), supplied teams with no-cost options to identify times for synchronous work. Initially, this CoP scheduled biweekly meetings at the same day and time of week to foster continuity of working sessions and to instill a shared sense of community. As the project progressed, a flexible approach to joint enterprise accommodated the evolving needs and tasks of the project (meeting frequency, working groups, iterative checking, etc), while respecting members’ autonomy and providing a predictable structure of interaction.

 With a team-based approach of approximately 17 active members, both synchronous and asynchronous forms of digital services were useful in organizing meetings, tasks, and sharing reflections. Online networking software including Google Hangouts, Zoom, and Skype provided communication options when expediency was needed. Synchronous chatting options were useful for live collaborations on manuscript production among working groups.

 A roadmap and task delegation forms were developed to assign roles and working groups for various aspects of the project (see Figure). For example, the qualitative component of the eDelphi study was divided into three working groups – one for each participant group (academic, industry and fellow) – with specific timelines and scheduled reminders for each team. This planning proved crucial in keeping project milestones during the data collection and analysis phases.

**Figure:**
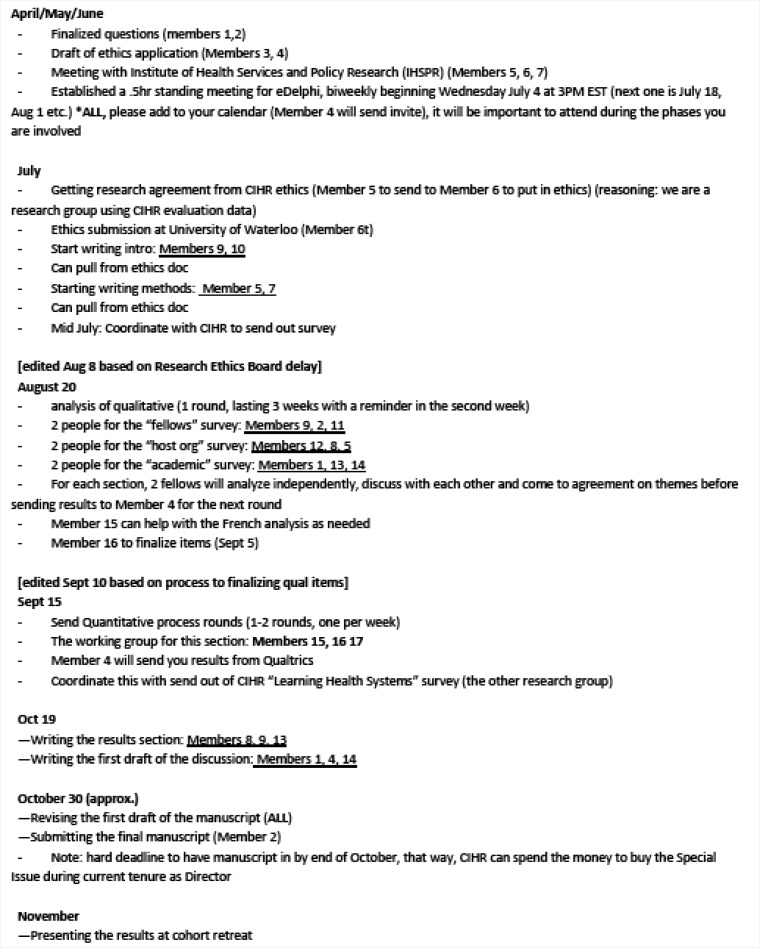
Project Roadmap and Task Delegation. Abbreviations: CIHR, Canadian Institute of Health Research; eDelphi, electronic Delphi.

####  (b) Communications and Social Interactions; Using Synchronous and Asynchronous Technologies to Develop Social and Professional Opportunities

####  Discussion and Approach

 Members of the CoP agreed upon a pragmatic approach that balanced insight and methodological simplicity. An eDelphi methodology replaced a resource-intensive study that would have required constant in-person engagement. Once the project charter was established by the steering group, group meetings took the form of working sessions. This meant the group co-wrote and made decisions in real time, incentivizing and invigorating synchronous participation. Online writing platforms (described below) promoted accountability by requiring members to sign-in and stimulate real-time participation by displaying the concurrent contributions of each participant. Participants were not reprimanded for not contributing, however the visibility of peer contributions served as a motivating factor for all fellows to be involved.

####  Peer to Peer Teaching/Idea Generation: Online Library Sharing, Database Sharing

 Throughout the collaboration, group meetings included dedicated time for routine check-ins. Meetings created space for members to clarify objectives, identify challenges or methodological tensions, and show and share expertise as peer mentors. As a result, both expertise and support were fluid and interchangeable. One of the major advantages of establishing this CoP involved reciprocal co-learning. During the early phases, following the initial brainstorm of ideas, members shared their knowledge of methods, analytical approaches and tools, and lessons learned related to impactful collaboration and knowledge translation and dissemination from previous projects.

For example, when the group decided to use an eDelphi methodology, training was needed for all members to understand the new approach. Co-learning was facilitated through informal online tutorials and by circulating and discussing the leading methodological literature. Using the Google Drive platform, a shared library was co-created to store resources and relevant materials. Co-learning activities continued during the analysis phase of the project, in which smaller working groups analyzed subsets of data (eg, qualitative and quantitative data sets). Google Classroom, a free web service for online learning through the creation, distribution and facilitation of online content, containing many services used in the CoP’s approach, may offer a better integrated approach that also connects to Google Suite (described below).^14^

####  (c) Output Platform; Online Editing and Storage Software

####  Writing

Several services provided by Google Suite, a package of integrated word processing, document collaboration, email, and online storage services, were employed throughout the CoP activities.^15^ Google Docs, an online word processing application embedded in Google Drive, has been described as a useful tool for collaborative work for university students^16^ with perceived potential for use by researcher populations.^17^ Google Docs allows for concurrent users to work on the same paper in real time and serves as an archive for previous versions stored online. In our work, the use of Google Docs allowed each member to contribute to meeting minutes, protocol development, ethics submissions, and the various phases of manuscript development.

####  Analytic Transparency 

Uniquely suited for data-driven, collaborative projects, GitHub was chosen as the platform to coordinate the analytic strategy. Following the principles of open science framework,^18^ we exposed the programming scripts and analytic reports in a public repository (https://github.com/andkov/hsif-2018-delphi), while keeping the survey responses private. Not only did this provide the opportunity for everyone to analyze the data, but it also increased the exchange of applied expertise.^19^ Featuring a concrete example of reproducible analytics in a collaborative project promoted sound data practices, which contributed to the joint development of the CIHR core competencies (data analysis, data management, evidence communication).

####  Online Security and Confidentiality

Use of Google Suite and other online services for data collection, cloud-based storage, and additional features raises issues related to security and confidentiality.^20^ The protection of users’ data poses a challenge as cloud computing expands to research collaborations.^21^ Although confidentiality and participant anonymity in the eDelphi study was managed by CIHR, the security of the CoP collaborative information was an important consideration. The choice of Google Suite services for document creation and collaboration was chosen primarily for features, familiarity, and convenience; an additional benefit was the data security provided. In fact, Google has taken important steps to preserve data integrity and user privacy and is recognized as a world leader in data security.^22^ The data in the eDelphi study was considered low risk as no participant or organizational identifiers were collected; however, future collaboratives that intend to use online cloud services will need to ensure the cloud services meets a high standard that addresses threats to security, privacy, and trust.^21^ It is important to note, that while paid alternatives to cloud collaboration may offer a higher degree of security (eg, Microsoft Teams) and enterprise support, it also creates a financial barrier for entry and may pose concerns to democratization of science.

###  3. Outcomes: Demonstrating Successes from Remote Collaboration

 The outcomes of this CoP may be categorized both in terms of academic success (tangible) and strengthening remote connections (intangible).

####  Academic Successes

Currently the most significant measurable success of this collaboration has been a peer-reviewed publication of the eDelphi study^7^ as part of a larger special issue in the Canadian health policy journal, *Healthcare Policy.* The study results were also presented at the annual HSIF cohort retreat, which included other HSIF fellows, as well as their academic and organizational mentors. Other research outputs resulting from remote collaboration among fellows are described elsewhere.^23,24^

####  Strengthening Remote Connections

 Intangible outcomes included the formation of personal and professional bonds among collaborating members of the HSIF CoP. The remote collaboration led to the establishment of local, geographically defined groups that held various in-person events and social activities. These types of unstructured opportunities were critical to reflecting on and retaining the knowledge acquired during the collaboration projects and the fellowship experience writ large. Furthermore, the remote collaboration eDelphi project and this subsequent perspective, has provided the authors with valuable experience to lead remote-based research projects within their own networks, and to foster further interdisciplinary collaboration. There is also an opportunity to mentor recent cohorts of the HSIF, and other early career researchers, on meaningful remote collaboration.

Reflecting on the experiences of the eDelphi study, remote collaboration provides a generative and feasible alternative when physical and geographic distances limit innovative and productive work. Beyond COVID-19, remote collaboration may equip research teams and learning health systems with tools and capacities to continue to think together and work apart effectively and productively. International collaboration provides career boosts as well an expansion of scientific inquiry and discovery.^25,26^ Remote collaboration provides an opportunity for researchers to create and maintain international collaboratives. Refinement of best practice international remote collaboration methods and outcomes is required to optimize participation and impact.^27^

## Conclusion

 The structure and process of our CoP supported efficiency and productivity in delivering on traditional research outputs and generated outcomes that expanded and enriched HSI fellows’ professional and social relationships. Effective use of remote collaboration using the CoP framework can positively contribute to emerging researchers’ career trajectories both by enhancing their networks and by creating a collective knowledge base when physical proximity is not possible. In a knowledge economy, the meaning and practice of working together has evolved; particularly in the health sector where the problems that bring diverse groups of researchers together are not only complicated but complex. Structuring remote collaboration using a CoP provides a mechanism that researchers can use to act swiftly and collectively to address emerging and unexpected challenges, such as working together under public health restrictions that include distancing during a pandemic, while maximizing the value and utility of existing accessible and cost-effective communication technologies. Beyond COVID-19, the resources and reflections presented offer a model that research teams could use to leverage digital technology in producing innovative and collaborative work, while also facilitating and sustaining meaningful long-distance working relationships.

 CoPs can play a crucial role in supporting remote collaboration under the conditions of scarcity and crisis becoming the ‘new normal’ in health systems. The value of digitally facilitated remote collaboration has been primarily assessed from the perspective of a ‘lack’ of health system tools and capacity. Our experience has illustrated that remote collaboration is not merely a feasible alternative; when framed by a CoP it offers a productive approach with positive outcomes. The innovation in this approach is not the technologies that facilitate it, but the ability of the approach to adapt and flex under changing conditions.

## Acknowledgements

 We would like to thank the Canadian Institute of Health Research and the Institute of Health Services and Policy Research for their assistance in the Health System Impact Fellowship and our career development.

## Ethical issues

 Not applicable.

## Competing interests

 Authors declare that they have no competing interests.

## Authors’ contributions

 Each author has contributed to the creation, data collection, drafting, and completion of the article.

## Authors’ affiliations

^1^Health Program, St. Francis Xavier University, Antigonish, NS, Canada. ^2^Women’s College Hospital Institute for Health System Solutions and Virtual Care, University of Toronto, Toronto, ON, Canada. ^3^Department of Sociology, St. Francis Xavier University, Antigonish, NS, Canada. ^4^University of Central Florida, Orlando, FL, USA. ^5^Canadian Autism Spectrum Disorder Alliance (CASDA), Toronto, ON, Canada.
